# Six Novel ATM Gene Variants in Sri Lankan Patients with Ataxia Telangiectasia

**DOI:** 10.1155/2020/6630300

**Published:** 2020-12-09

**Authors:** D. Hettiarachchi, Hetalkumar Panchal, B. A. P. S. Pathirana, P. D. Rathnayaka, A. Padeniya, P. S. Lai, V. H. W. Dissanayake

**Affiliations:** ^1^Human Genetics Unit, Faculty of Medicine, University of Colombo, Colombo, Sri Lanka; ^2^Post Graduate Department of Bioscience, Sardar Patel University, Vallabh Vidyanagar, Gujarat, India; ^3^Lady Ridgway Hospital for Children, Colombo, Sri Lanka; ^4^Department of Paediatrics, Yong Loo Lin School of Medicine, National University of Singapore, Singapore

## Abstract

**Introduction:**

Ataxia telangiectasia is a rare genetic condition with an estimated prevalence of 1 in 40,000–100,000 live births. This condition predominantly affects the nervous and immune systems. It is characterized by progressive ataxia beginning from early childhood. The neurological deficit associated with this condition affects one's balance, coordination, walking, and speech and can be accompanied by chorea, myoclonus, and neuropathy. They may also have ocular telangiectasias and high levels of blood alpha-fetoprotein (AFP). The ataxia telangiectasia mutated gene (ATM) is associated with this condition and codes for the ATM protein which is a phosphatidylinositol 3-kinase. This gene occupies 150 kb on chromosome 11q22–23 and contains 66 exons encoding a 13 kb transcript. ATM is a relatively large protein with a molecular weight of 350 kDa and 3,056 amino acids.

**Methods:**

Four patients of Sri Lankan origin presenting with features suggestive of ataxia telangiectasia were referred to our genetics center for specialized genetic counseling and testing. Whole-exome sequencing followed by Sanger sequencing was used to confirm the candidate variants. Protein modeling and genotype to phenotype correlation was performed in the identified variants.

**Results:**

We observed 6 novel ATM gene variants in four patients with ataxia telangiectasia. The identified variants are as follows: homozygous c.7397C > A (p.Ala2466Glu) and c.510_511delGT (p.Tyr171fs) and compound heterozygous c.5347_5350delGAAA (p.Glu1783fs), c.8137A > T (p.Arg2713^*∗*^) and c.1163A > C (p.Lys388Thr), and c.5227A > C (p.Thr1743Pro). Variant analysis was followed by modeling of the native and altered protein structures.

**Conclusion:**

We report novel *ATM* gene variants that have implications on the molecular diagnosis of ataxia telangiectasia.

## 1. Introduction

The ataxia telangiectasia mutated gene (ATM) codes for the ATM protein which is a phosphatidylinositol 3-kinase that responds to DNA damage. It is a key substrate in DNA repair and cell cycle control [1, 2]. ATM gene occupies a 150 kb space on chromosome 11q22–23 which contains 66 exons encoding a transcript of 13 kb. ATM is a relatively large protein with a molecular weight of 350 kDa containing 3,056 amino acids. Many ATM transcripts have been identified which share almost the same open reading frames of 9.2 kb and exhibit numerous 5′-untranslated regions (5′-UTRs) which are formed by complex alternative splicing and several alternative 3′-UTRs [3, 4]. Ataxia telangiectasia (A‐T; MIM# 208900) is an autosomal recessive genetic disorder caused by biallelic inactivation of the ATM gene [5]. This condition is primarily characterized by cerebellar degeneration dominated by gradual cerebellar cortical atrophy which results in progressive ataxia beginning from early childhood. Neurological deficit affects one's balance, coordination, walking, and speech and can be accompanied by chorea, myoclonus, and neuropathy as well as telangiectasias in the eyes and sometimes on the facial skin. Thymic degeneration, immunodeficiency, recurrent sino-pulmonary infections, retarded somatic growth, premature aging, gonadal dysgenesis, predisposition to lymphoreticular malignancies, and acute sensitivity to ionizing radiation are some of the other associated features. It is reported that AT carriers are suspected to be cancer-prone which is often due to genomic instability or DNA damage response syndrome. The worldwide prevalence of AT is estimated to range from 1 in 40,000 and 1 in 100,000 live births [6, 7]. In this study, we describe 6 novel ATM gene variants in 4 patients with clinical features of ataxia telangiectasia using next generation sequencing.

## 2. Methods

### 2.1. Selection of Patients

Four patients who were clinically diagnosed with AT were referred for genetic testing and counseling to the Human Genetics Unit, Faculty of Medicine, the University of Colombo. Written informed consent was obtained from the patient's parents following a protocol approved by the Ethics Review Committee, Faculty of Medicine, University of Colombo, Sri Lanka.

### 2.2. Sample Collection and DNA Extraction

Venous blood samples in EDTA tubes were obtained from the patients and extraction of genomic DNA from peripheral blood leukocytes was performed using a QIAamp DNA Mini Kit according to the manufacturer's protocol [8].

### 2.3. Next Generation Sequencing

The patients underwent whole-exome sequencing on an Illumina HiSeq platform followed by library preparation using the Agilent SureSelect Human All Exon + UTR Kit according to the manufacturer's protocol [9]. Genetic analysis of the paired-end sequencing data was performed using an in-house bioinformatics pipeline. FASTQ files were mapped with the (GRCh37/hg19) human reference sequence using the BWA‐mem algorithm and Genome Analysis Tool Kit (GATK). The annotation of the VCF file was performed using SNP-eff with Refseq, clinical databases, and population frequency databases available online [10–12]. All sequence variants were confirmed by visual inspection of alignments, followed by Sanger sequencing.

Genes that matched the AT phenotype (ATM, MRE11A, and NBS1) and other genes associated with cerebellar ataxia (RAD50, RNF168, APTX, SETX, TWNK, POLG, and ABCB7) were analyzed in all four probands. They were classified according to standard ACMG guidelines (https://www.acgs.uk.com/media/11631/uk-practice-guidelines-for-variant-classification-v4-01-2020.pdf). *In-silico* functional prediction tools were employed to study the pathogenicity of the identified variants.

### 2.4. Protein Modeling

Modeling of the native and truncated structure of the ATM protein was performed for each identified variant using the SWISS-MODEL server (http://swissmodel.expasy.org). The mRNA sequence of serine-protein kinase/ataxia telangiectasia mutated ATM protein was retrieved in FASTA format from GenBank with GenBank ID:  U33841.1.

## 3. Results

### 3.1. Case Presentation

**Figure 1:**
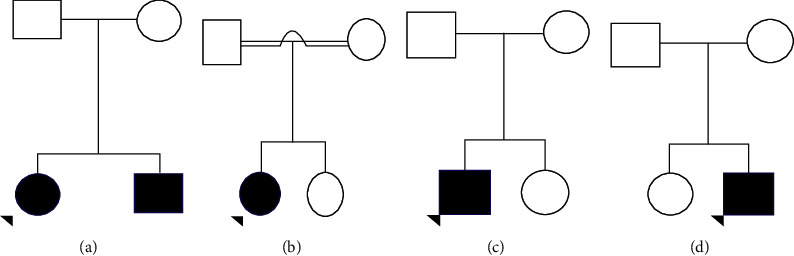
Pedigrees of the patients diagnosed with AT following whole-exome sequencing.

Case 1 was a 6-year-old girl representing one of the two affected siblings of a nonconsanguineous marriage (Figure 1(a)). She was born at term without complications following a normal pregnancy. She developed cerebellar ataxia with asynergy (lack of coordination between muscles) and spasticity (increase in tendon reflexes) around 3 years of age. Her brain MRI showed no abnormalities. Her alpha-fetoprotein (AFP) level was 172.2 *μ*g/ml (elevated). However, other blood investigations were within the normal range. She was clinically diagnosed with AT. She did not have any ocular telangiectasias or immunological features. Her brother also showed similar signs of cerebellar ataxia during his early years. Both parents were unaffected. However, with age, their symptoms gradually reduced. Currently, the proband suffers from minimal writing and walking difficulty and her brother is almost asymptomatic.

Case 2 was a 9-year-old girl born to consanguineous parents (Figure 1(b)). She was born without complications at term following a normal pregnancy. Her sister and parents are unaffected. She was diagnosed with epilepsy at 2 years of age and developed difficulty in walking and other cerebellar signs around 7 years of age. Currently, she is experiencing frequent syncopal attacks. She also has dysarthric speech, dystonia, tremors, ocular telangiectasias and suffers from recurrent respiratory tract infections. Her AFP levels are between 200–300 *μ*g/ml. Her other blood parameters are within normal range and there are no abnormalities in her brain MRI scan. However, the severity of her condition has progressed over the years.

Case 3 was a 7-year-old boy born at term to nonconsanguineous parents following a normal uneventful pregnancy (Figure 1(c)). He showed signs of cerebellar ataxia and difficulty in walking during early childhood. At the age of 2 years, he was clinically diagnosed with AT. His blood AFP level was elevated at 320.1 *μ*g/ml. His other blood parameters and MRI of the brain were normal. Currently, he suffers from dysarthria, dystonia, ocular telangiectasias, and recurrent respiratory tract infections. His parents and sibling (sister) are asymptomatic and healthy.

Case 4 was a 24-year-old male born to nonconsanguineous parents following an uneventful pregnancy. He presented with late-onset ataxia, dysarthria, dystonia, and difficulty in walking. He has no ocular telangiectasias or immunological features. He also shows signs of dystonia. His symptoms have not shown any progression or improvement for the past 2 years. His blood AFP level is only mildly elevated 49 *μ*g/ml with other blood parameters within the normal range. His MRI brain is normal and his parents and sibling (sister) are unaffected (Figure 1(d)).

Genotype to phenotype characteristics along with alpha-fetoprotein (AFP) levels, family history of neurological, immunological disorders, and cancers are described in Table 1.

### 3.2. Variant Identification

We observed 6 novel ATM gene variants in four patients with AT. The variants were as follows: homozygous c.7397C > A (p.Ala2466Glu) and c.510_511delGT (p.Tyr171fs) and compound heterozygous c.5347_5350delGAAA (p.Glu1783fs), c.8137A > T (p.Arg2713^*∗*^) and c.1163A > C (p.Lys388Thr), and c.5227A > C (p.Thr1743Pro). These variants were confirmed using Sanger sequencing.

### 3.3. Comparison of Variants with AT Phenotype

We compared the phenotypic severity with the functional predictions observed for each variant (Table 1). *In-silico* tools were employed to derive the altered protein structure.

### 3.4. Pathogenicity of the Variants

The variants observed in this study were predicted to be damaging when analyzed using functional prediction software tools such as Mutation Taster, disease mutation; Provean, deleterious; Polyphen2, damaging; SIFT  damaging. Variants predicted as damaging are likely to be pathogenic.

## 4. Discussion and Conclusion

**Figure 2:**
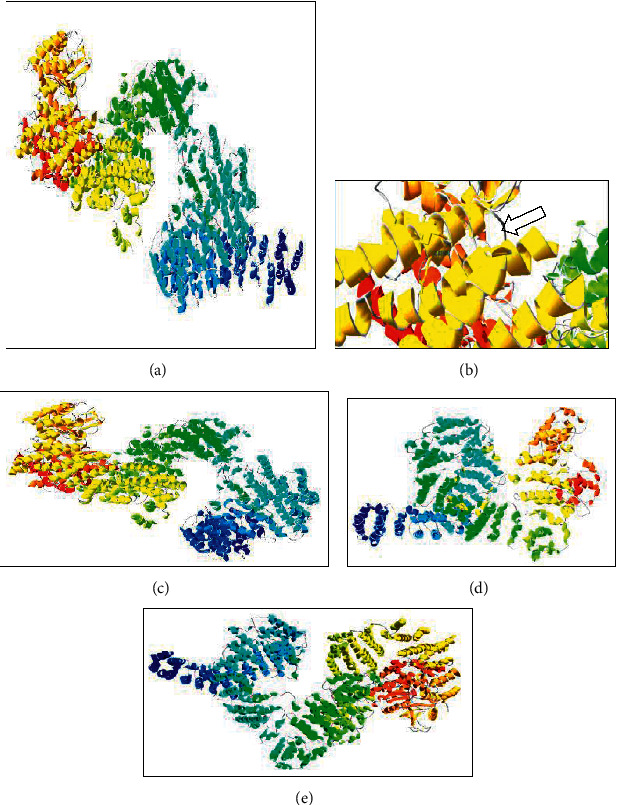
Protein modeling. (a) Normal ATM protein, (b) ATM protein structure with Glu at 2466 in helix, (c) structure of the protein with a missing helix, (d) structure of the ATM protein synthesized after deletion of GAAA from normal ATM mRNA at 5347_5350,and (e) 3D structure unaltered.

Ataxia telangiectasia is a clinically heterogeneous neurological condition resulting from the loss of ATM protein which is a relatively large protein (350 kDa). It comprises of 3056 amino acids (Figure 2(a)) and belongs to the family of phosphatidylinositol 3-kinase-like protein kinases [13]. It was observed that severe forms were associated with a total loss of ATM protein while milder, slow-progressing, or late-onset forms of AT were caused by nontruncating variants such as missense or splice site variants which rendered some residual ATM kinase activities within the cells [14]. To date, over 1700 variants in the ATM gene have been reported, and among them, over 800 variants have shown to be associated with the AT phenotype (http://www.hgmd.cf.ac.uk/ac/gene.php?gene=ATM). The majority of the pathogenic variants resulting in the total loss of the ATM protein account for about 75% of the AT phenotype [15, 16]. Molecular cloning studies targeted at the cDNA spanning the complete open reading frame of the ATM gene and the ATM protein shows significant sequence similarities to several large proteins across species, e.g., *Drosophila*, yeast, and mammals, as they all share a similar PI 3-kinase domain. Mutations in their genes confer a variety of phenotypes with features similar to those observed in human cells affected with AT [13]. Hence, those missense variants residing over highly conserved domains across species are more likely to be pathogenic.

In our study, we have observed a similar correlation between the type of variants and phenotypic severity. Thus, in Case 1, homozygous missense variant (c.7397C > A) was located in exon 48. This region has 11 overlapping transcripts; studies have shown the 48^th^ exon, which spans 114 bp, affects the mitochondrial function related to ataxia telangiectasia [17, 18]. It was observed that at 2466^th^ position the mutated protein had a substitution in one amino acid (i.e., Ala to Glu), while the rest of the amino acids remained unchanged (Figure 2(b)). It was also observed that the change in a single amino acid did not affect the secondary and tertiary structures of the protein. However, this change was situated in a highly conserved protein domain across species (Table 2).

In Case 2, homozygous frameshift variant (c.510_511delGT) was located on exon 3 which spans 113 bps. We observed the amino acids coded by mutated mRNA remained unchanged up to the 510^th^ position. However, the deletion of G and T nucleotides at the 510^th^ and 511^th^ positions respectively results in a frameshift in the remaining mRNA sequence. This change caused the formation of a premature stop codon immediately after the 511^th^ position, thus resulting in the synthesis of a truncated protein with only 2708 amino acids compared to the 3056 amino acids in the wild type protein (Figure 2(c)). The frequent occurrence of truncated mutations in AT patients suggests that the PI 3-kinase domain at the 3′ end of the gene is indispensable, therefore resulting in a more severe phenotype. Several studies have shown that truncating variants lead to a more severe early-onset AT [19].

In Case 3, a compound heterozygous variant was observed. One was a frameshift (c.5347_5350delGAAA) and the other was a missense variant (c.8137 A > T). c.5347_5350delGAAA resides on exon 33 which spans 96 bps. Deleted GAAA nucleotides from 5347–5350 positions in mRNA sequence resulted in the formation of a stop codon immediately after the 5370^th^ position and a truncated protein of 1790 amino acids was synthesized (Figure 2(d)). The second variant in this patient c.8137 A > T resides in exon 52 spanning 159 bps and has 12 overlapping transcripts. This variant leads to a premature stop codon at p.Arg2713∗ position further leading to the truncation of the ATM protein. In previous studies, similar compound, heterozygous variants have shown phenotypic heterogeneity and late-onset AT [20]. However, this was mainly observed when variants reside outside the PI 3-kinase domain (aa 2857–2915). This suggests that missense mutations outside the catalytic domain may result in residual ATM activity. However, we observed that this hypothesis was subjective as frameshift variants residing outside this domain also resulted in a severe early onset form of AT.

In Case 4, there was a compound heterozygous missense variant denoted as c.1163A > C and c.5227A > C residing on exon 3 and 32, respectively. As per our findings, we changed the nucleotides at 1163A > C and 5227A > C manually in the mRNA sequence. In the translated protein sequence, it was found that there is a substitution of Lys > Thr at the 388^th^ position and Thr > Pro at the 1743^rd^ position but the remaining amino acids were unchanged. These compound heterozygous variants do not appear to have a marked impact on the 3D structure of the protein. However, the second amino acid change (p.Thr1743Pro) appears to occur in a domain that is highly conserved across species (Table 2). This patient had a late-onset milder form. In addition to variants exhibiting milder forms of AT, other clinical features such as dystonia have also been described along with the late presentation [19].

Variant detection in the ATM gene has advanced since the advent of next generation sequencing as it is a relatively large gene with 63 exons and no documented hotspot regions [21]. Current studies are predominantly based on Caucasian populations [22, 23]. There are not many studies conducted in southeast Asian patients and none describing the genotype of Sri Lankan AT patients. Identifying new disease-causing variants is becoming increasingly important for genetic testing and it is leading to a significant change in the scale and sensitivity of molecular genetic analysis in AT. The hope of a new therapy to reverse the effects of the absence of a functional ATM kinase is on the horizon [24]. In keeping with already published literature, the results from our study showed that truncating variants resulted in an earlier onset disease with increased severity and vice versa for missense variants where some residual activities of the ATM kinase would be present. In conclusion, we have demonstrated the 6 novel ATM gene variants resulting in AT in Sri Lankan patients.

### 4.1. Limitations

The main limitation of this study is the sample size. We would like to expand WES to other patients who are clinically diagnosed with AT. Furthermore, for missense variants, functional studies can complement *in-silico* analysis to further decipher the exact effect on ATM kinase.

### 4.2. Future Work

It should be possible to detect AT shortly after birth and early referral for genetic studies for confirmation should take precedence, as it will enable individualized patient interventions and genetic counseling for the family members, especially those who are carriers for ATM gene variants. They can also be followed up for neurological, immunological disorders and cancers, as they will have a higher risk of developing malignancies.

## Figures and Tables

**Table 1:** Comparison of mutation positions of the cases and its effect on protein sequence and phenotypic severity.

Case	Novel variants	Current evidence on the effect of the variant	Effect on ATM	Protein structure	AFP level (<10 *μ*g/ml)	Phenotypic severity
1	c.7397C > A homozygous, missense	This variant is located in exon 48 of the ATM gene. Variants in this region overlaps 11 transcripts which code for mitochondrial fission factor interactor, a protein required for mitochondrial function.	p.Ala2466Glu	This single amino acid change did not affect the secondary and tertiary structures.	172.2	Mild ataxia; no family history of neurological, immunological disorders, or cancers

2	c.510_511delGT, homozygous, frameshift	This variant is located in exon 3 and has 14 overlapping transcripts. Frameshift variants lead to truncation of the ATM protein. This type of variants is associated with a more severe phenotype.	p.Tyr171fs	A partial protein was synthesized with only 2708 amino acids (wild type protein had 3056 amino acids)	200–300	Severe phenotype with multisystem involvement and recurrent infections; no family history of neurological, immunological disorders, or cancers

3	c.5347_5350delGAAA and c.8137A > T compound heterozygous	A deletion in the exon 33 of the ATM gene was observed. There are 8 overlapping transcripts in this region. The first variant leads to the formation of a truncated ATM protein and the second missense variant leads to a premature stop codon	p.Glu1783fs p.Arg2713^*∗*^	The synthesized protein had only 1790 amino acids.	320.1	Severe early-onset ataxia, dysarthria, dystonia, and recurrent respiratory tract infections; no family history of neurological, immunological disorders, or cancers

4	c.1163A > C and c.5227A > C compound heterozygous	The first missense variant resides on exon 8; the second variant resides on exon 32 which is also a missense variant; missense variants were observed to result in a milder phenotype	p.Lys388Thr p.Thr1743Pro	The secondary and the tertiary structures of the protein remained unchanged	49	Mild late-onset AT phenotype; no family history of neurological, immunological disorders, or cancers

**Table 2:** Conservation of the mutated domain across species in comparison to Cases 1 and 4.

Proband in Case 1	QRELELDEL *E* LRALKEDRKRF	Proband in Case 4	NTLVEDCVKVRSAAV *P* CL
*Homo sapiens*	QRELELDEL *A* LRALKEDRKRF	*Homo sapiens*	NTLVEDCVKVRSAAV *T* CL
Human		Human	

*Mus musculus*	QRELELDEC *A* LRALREDRKRF	*Mus musculus*	NTLVEDSVKIRSAAA *T* CL
House mouse		House mouse	

*Rattus norvegicus*	QRELELDEC *A* LRALKEDRKRF	*Rattus norvegicus*	NTLVEDSVKIRSAAA *T* CL
Norway rat		Norway rat	

*Sus scrofa*	QRELELDEG *A* LRALKKDRKR	*Sus scrofa*	STLVEDCVKVRSAAV. *T* CL
Pig		Pig	

*Canis lupus familiaris*	QRELELDEC *A* LRALKEDRKRF	*Canis lupus familiaris*	NTLVEDCVKVRSAAV. *T* CL
Dog		Dog	

*Macaca mulatta*	QRELELDEL *A* LHALKEDRKRF	*Macaca mulatta*	NTLVEDCVKVRAAAV *T* CL
Rhesus monkey		Rhesus monkey	

*Xenopus tropicalis*	QRELELDEC *A* ILALREDRKRF	*Xenopus tropicalis*	NTLVEDCVKVRSAAV. *T* CL
Tropical clawed frog		Tropical clawed frog	

*Pan troglodytes*	QRELELDEL *A* LRALKEDRKRF	*Pan troglodytes*	NALTDHCIQVRSAAA. *T* CL
Chimpanzee		Chimpanzee	

## Data Availability

Data are not available in the public domain; they will be shared on request from interested parties, this is to maintain patient confidentiality.

## Abbreviations

ATM: Ataxia telangiectasia mutated
AT: Ataxia telangiectasia
WES: Whole-exome sequencing
cDNA: Complementary DNA
SPDBV: Swiss-PdbViewer.

## References

[1] Savitsky K., Bar-Shira A., Gilad S. (1995). A single ataxia telangiectasia gene with a product similar to PI-3 kinase. *Science*.

[2] McKinnon P. J. (2004). ATM and ataxia telangiectasia. *EMBO Reports*.

[3] Savitsky K., Uziel T., Gilad S. (1997). Ataxia-telangiectasia: structural diversity of untranslated sequences suggests complex post-transcriptional regulation of ATM gene expression. *Nucleic Acids Research*.

[4] Rotman G., Yosef S. (1998). ATM: from gene to function. *Human Molecular Genetics*.

[5] Fiévet A., Bellanger D. (2019). Functional classification of ATM variants in ataxia‐telangiectasia patients. *Human Mutation*.

[6] Rothblum-Oviatt C., Wright J., Lefton-Greif M. A., McGrath-Morrow S. A., Crawford T. O, Lederman H. M. (2016). Ataxia telangiectasia: a review. *Orphanet Journal of Rare Diseases*.

[7] Lavin M. F., Shiloh Y. (1997). The genetic defect in ataxia-telangiectasia. *Annual Review of Immunology*.

[8] Qiagen.Com, 2020, https://www.qiagen.com/ch/resources/download.aspx?id=62a200d6-faf4-469b-b50f-2b59cf738962&lang=en

[9] Agilent.Com, 2020, https://www.agilent.com/cs/library/datasheets/public/SureSelect%20V6%20DataSheet%205991-5572EN.pdf

[10] Index of/pub/Clinvar/Vcf_Grch37”. Ftp.Ncbi.Nlm.Nih.Gov, 2020, https://ftp.ncbi.nlm.nih.gov/pub/clinvar/vcf_GRCh37/

[11] The Genome aggregation database (gnomad) | gnomad blog”. Gnomad.Broadinstitute.Org, 2020, https://gnomad.broadinstitute.org/blog/2017-02-the-genome-aggregation-database/

[12] Exac allele frequency of pathogenic clinvar variants - dave tang’s blog”. Dave Tang’s Blog, 2020, https://davetang.org/muse/2017/01/30/exac-allele-frequency-pathogenic-clinvar-variants/

[13] Van Os N. J. H., Jansen A. F. M., Van Deuren M. (2017). Ataxia-telangiectasia: immunodeficiency and survival. *Clinical Immunology*.

[14] Verhagen M. M. M., Last J. I., Hogervorst F. B. L. (2012). Presence of ATM protein and residual kinase activity correlates with the phenotype in ataxia-telangiectasia: a genotype-phenotype study. *Human Mutation*.

[15] Jacquemin V., Rieunier G., Jacob S. (2012). Underexpression and abnormal localization of ATM products in ataxia telangiectasia patients bearing ATM missense mutations. *European Journal of Human Genetics*.

[16] Pourahmadiyan A., Alipour P., Golchin N., Tabatabaiefar M. A. (2020). Next generation sequencing reveals a novel pathogenic variant in the ATM gene. *International Journal of Neuroscience*.

[17] Pallardó F. V., Lloret A., Lebel M. (2010). Mitochondrial dysfunction in some oxidative stress-related genetic diseases: ataxia-telangiectasia, down syndrome, fanconi anaemia and werner syndrome. *Biogerontology*.

[18] Nakamura K., Du L., Tunuguntla R. (2012). Functional characterization and targeted correction of ATM mutations identified in Japanese patients with ataxia-telangiectasia. *Human Mutation*.

[19] Gilad S., Chessa L., Khosravi R. (1998). Genotype-phenotype relationships in ataxia-telangiectasia and variants. *The American Journal of Human Genetics*.

[20] Saviozzi S., Saluto A. (2002). A late onset variant of ataxia-telangiectasia with a compound heterozygous genotype, A8030G/7481insA. *Journal of Medical Genetics*.

[21] Martin‐Rodriguez S., Calvo-Ferrer A., Ortega-Unanue N., Samaniego-Jimenez L., Sanz-Izquierdo M. P., Bernardo-Gonzalez I. (2019). Two novel variants in the ATM gene causing ataxia‐telangiectasia, including a duplication of 90 kb: utility of targeted next‐generation sequencing in detection of copy number variation. *Annals of Human Genetics*.

[22] Shiloh Y., Ziv Y. (2013). The ATM protein kinase: regulating the cellular response to genotoxic stress, and more. *Nature Reviews Molecular Cell Biology*.

[23] Suspitsin E., Sokolenko A., Bizin I. (2020). ATM mutation spectrum in Russian children with ataxia-telangiectasia. *European Journal of Medical Genetics*.

[24] Ovchinnikov D. A., Withey S. L., Leeson H. C. (2020). Correction of ATM mutations in iPS cells from two ataxia-telangiectasia patients restores DNA damage and oxidative stress responses. *Human Molecular Genetics*.

